# Supplementary material for: Anti-Alopecia Activity of Alkaloids Group from Noni Fruit against Dihydrotestosterone-Induced Male Rabbits and Its Molecular Mechanism: In Vivo and In Silico Studies
Source: Pharmaceuticals (Basel). 2022 Dec 14;15(12):1557. doi: 10.3390/ph15121557 (PMC9784383; doi:10.3390/ph15121557)

Anti-alopecia Activity of Alkaloids Group from Noni Fruit Against Dihydrotestosterone Induced Male Rabbits and Its Molecular Mechanism: *In vivo* and *In silico* Studies

Laila Susanti[1,2*], Resmi Mustarichie[1*], Eli Halimah[3], Dikdik Kurnia[4], Andi Setiawan[5] and Yustinus Maladan[6]

Supplementary Figures

Supplementary Figures S1. A representative photograph of rabbits shows the extent of alopecia after 17 days of DHT induction.

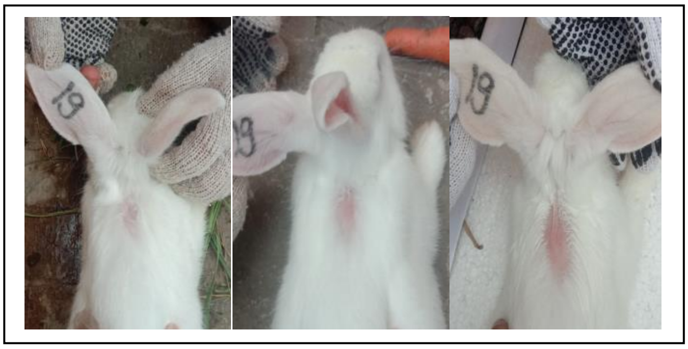

Supplementary Figures S2-S7. The MS chromatogram of FEA-3 sub-fraction

Figure S2. Mass spectrum of (E)-3-(1H-Indol-3-yl)acrylonitrile

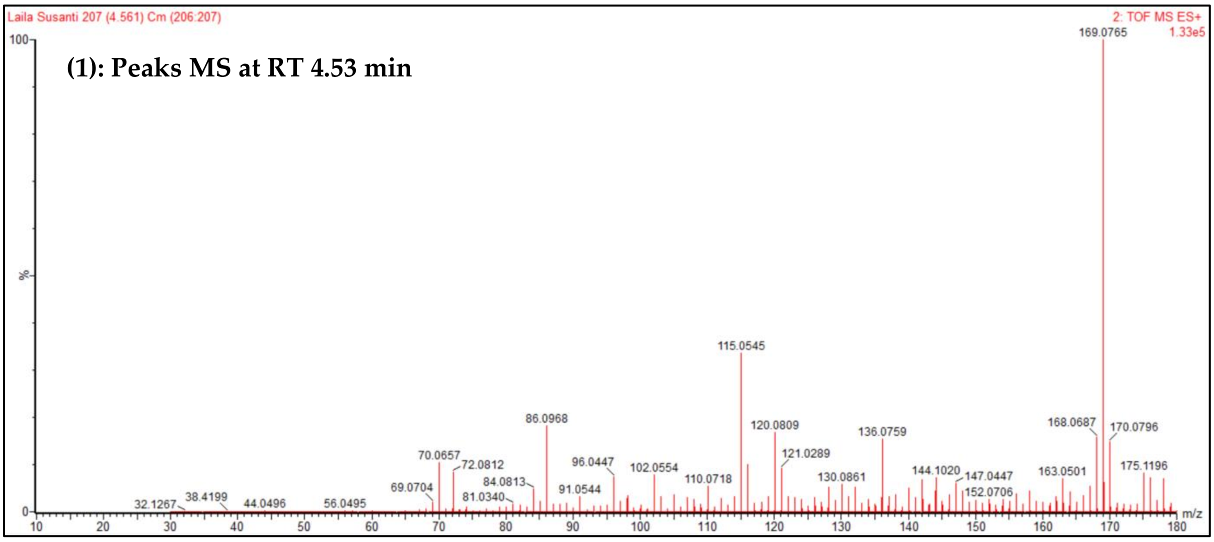

Figure S3. Mass spectrum of 1-[4-(2-Hydroxyethyl)-1-piperazinyl]-3-[(2-isopropyl-5-methylcyclohexyl)oxy]-2-propanol

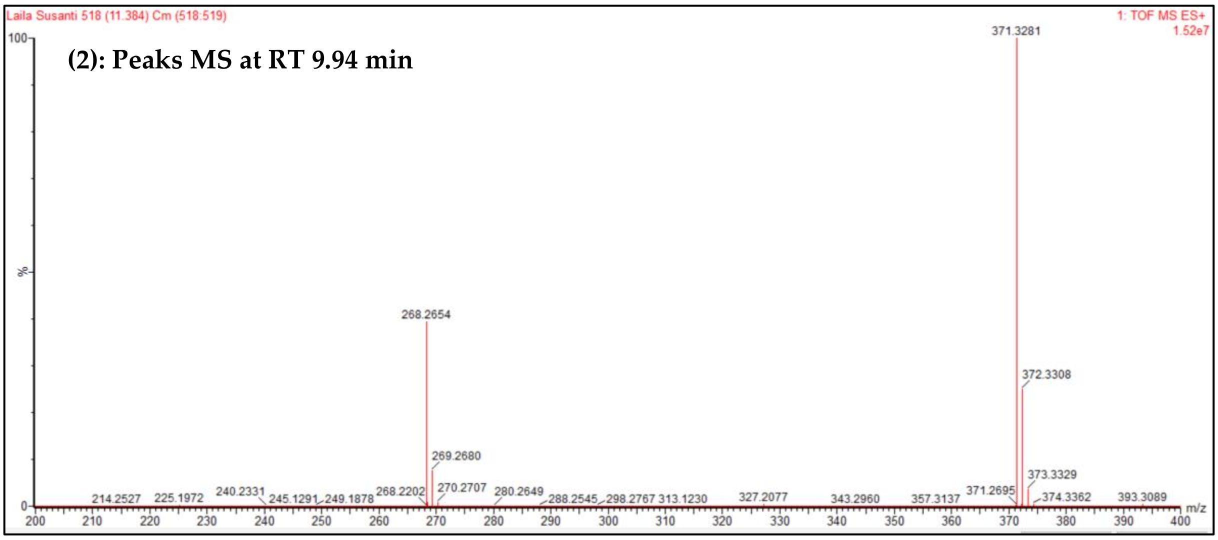

Figure S4. Mass spectrum of 11-[(1-Hydroxy-2,2,6,6-tetramethyl-4-piperidinyl)(methyl)amino]undecanoic acid

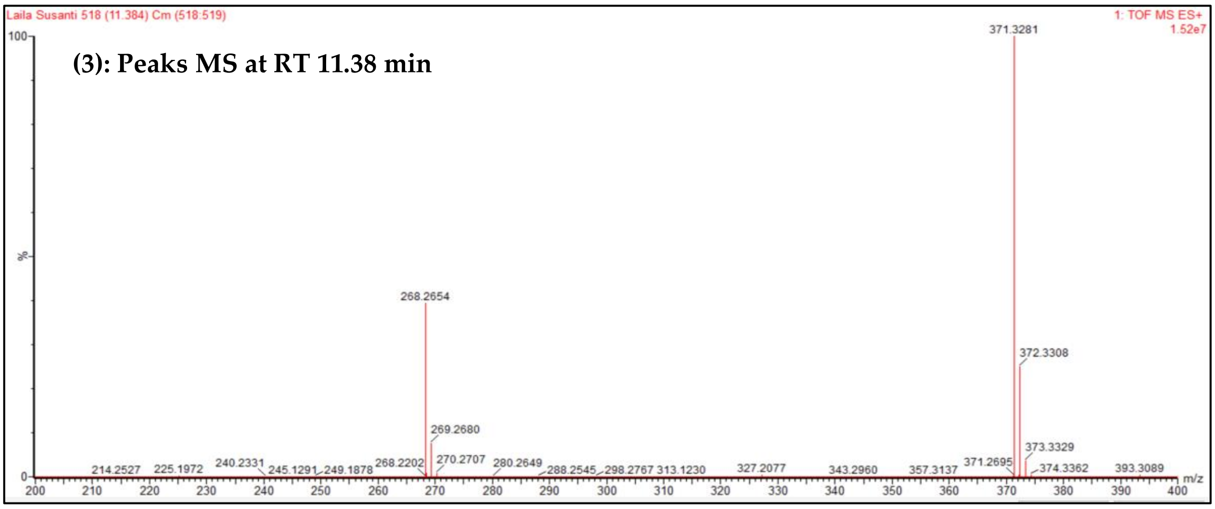

Figure S5. Mass spectrum of 2-Methyl-2-propanyl [(3S,4S,6S)-4-hydroxy-6-tridecyl-3-piperidinyl]carbamate

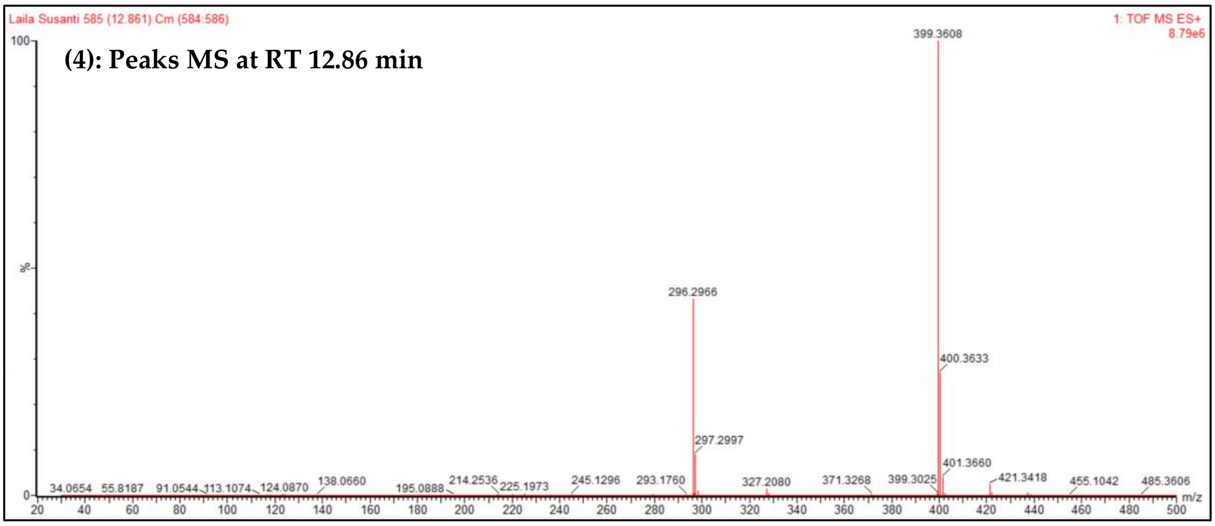

Figure S6. Mass spectrum of 3-[3-(Dimethylamino)propyl]-5-[(hexadecyloxy)methyl]-1,3-oxazolidin-2-one

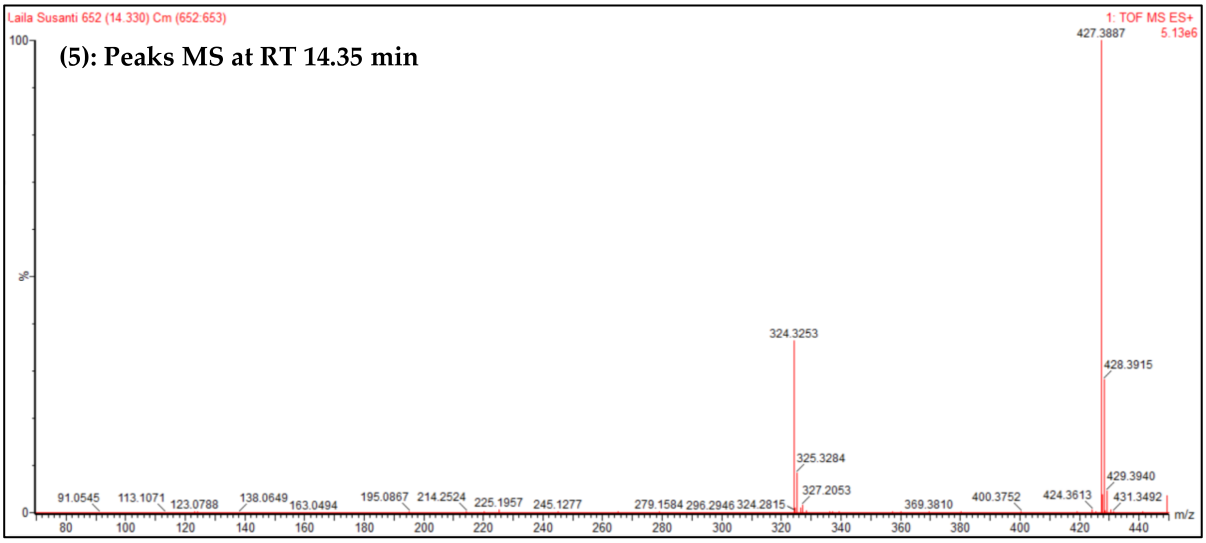

Figure S7. Mass spectrum of 2,2',2'',2'''-({4,8-Bis[(3,4-dimethoxybenzyl)amino]pyrimido[5,4-d]pyrimidine-2,6-diyl}dinitrilo)tetraethanol

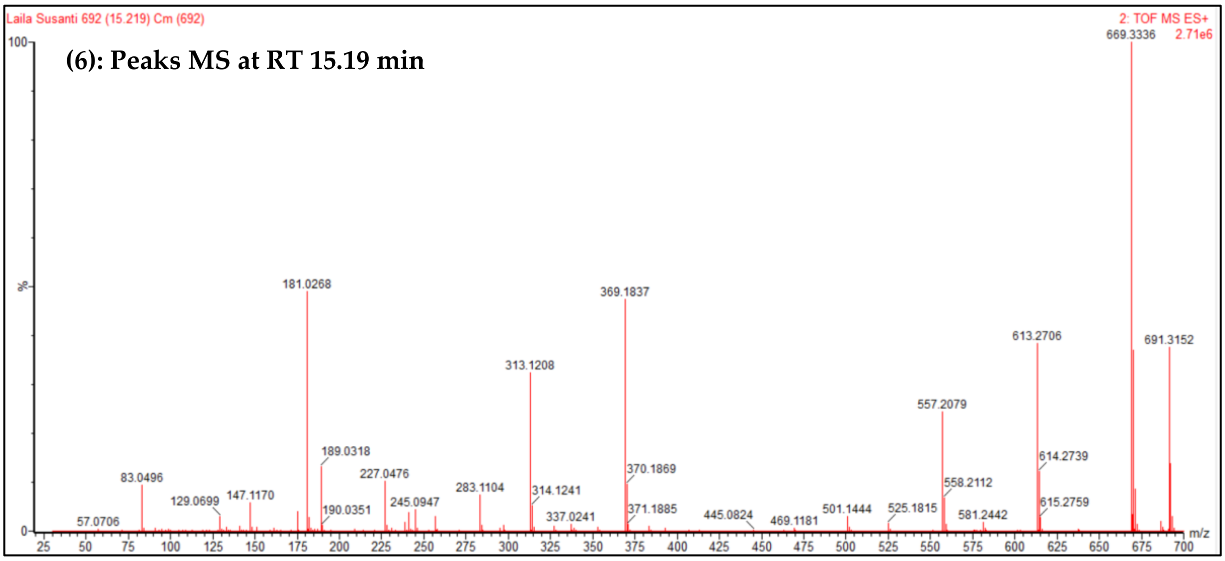

Supplement: Supplementary file 1 [file pharmaceuticals-15-01557-s001.zip › pharmaceuticals-2070965-supplementary.pdf]
